# The lavender purge

**DOI:** 10.1002/jia2.26509

**Published:** 2025-05-24

**Authors:** Kenneth H. Mayer

**Affiliations:** ^1^ The Fenway Institute, Fenway Health Boston Massachusetts USA; ^2^ Department of Medicine Harvard Medical School and Beth Israel Deaconess Medical Center Boston Massachusetts USA

1

In May 1933, the Nazis looted Berlin's Institute for Sexual Science, founded by the highly regarded researcher, Dr. Magnus Hirschfeld, destroying his archives in a book burning. Hirschfeld recognized that although most humans are cisgender heterosexuals, sexual and gender minorities have existed throughout human history, and their behaviours and identities needed to be considered as part of the human continuum [1]. Sexual orientation, gender identity and sexual behaviours do not necessarily overlap in uniform and predictable ways; each domain may vary throughout the life course, and may be expressed in different ways in diverse cultures.

Over the past few months, the Trump Administration has issued Executive Orders from the President that have promulgated policies that are detrimental to the lives of sexual, gender, racial and ethnic minorities, as well as people living in low‐ and middle‐income countries who have been affected by HIV and other major public health challenges. One manifestation of these orders has been that hundreds of researchers have received notices from several United States government agencies, including the National Institutes of Health (NIH, the major funder of health research in the United States), as well as the Centers for Disease Control and Prevention (the major funder of public health programmes in the United States), that stated: “This award no longer effectuates agency priorities. Research programs based primarily on artificial and nonscientific categories, including amorphous equity objectives, are antithetical to the scientific inquiry, do nothing to expand our knowledge of living systems, provide low returns on investment, and ultimately do not enhance health, lengthen life, or reduce illness.” The Trump Administration terminated studies that it deemed to be too focused on “gender ideology” and “diversity, equity and inclusion.” The spate of NIH grant terminations for research studies that addressed sexual behaviour, gender identity, discrimination and health equity among other proscribed topics (e.g. climate change) is reminiscent of earlier regressive periods.

The studies that were terminated had undergone the rigorous process of peer review, wherein applications were examined by external experts, scored and discussed with NIH project officers who then determined which projects were fundable after discussions with their institutes’ leadership. The process is rigorous and highly competitive. Only 10–20% of applications that are submitted for consideration ultimately get funded, so the terminations eliminated research that was deemed to be highly promising after careful assessment. The same rigor applied to the programmes funded by the CDC, USAID and PEPFAR, resulting in the abrupt elimination of needed services for millions.

The recent NIH, PEPFAR and USAID terminations have not been based on objective evidence. A prime example of how the Trump Administration actions have been predicated on bias and not objective measures can be seen in their approach to AIDS, given that HIV research has turned a lethal disease into treatable, and preventable, health condition—but one requiring ongoing access to medication and functional healthcare systems. Despite considerable progress, in the absence of an effective cure or vaccine, almost 1.4 million people acquired HIV last year [2], and these numbers will invariably increase if evidence‐based interventions and new research are not supported. Sexual and gender minority people constitute the majority of people living with, and at risk for, HIV in the United States and bear a high disease burden globally, so the termination of hundreds of HIV‐focused grants will disproportionately affect LGBTQ+ people and other key and priority populations throughout the world. The U.S. governmental research grants that have been terminated include clinical trials and observational studies that are conducted in multinational networks, so the impact of these decisions has immediate global implications. Programmes funded by PEPFAR and USAID have provided essential medications and services for HIV treatment and prevention for millions of people in low‐ and middle‐income countries, including substantial numbers of sexual and gender minority people. These programmes addressed societal homophobia and transphobia in many countries, so their elimination is likely to result in erosions of civil society protections for sexual and gender minority populations.

Repairing the damage from these ill‐considered decisions will require many years—which is similar to the decades it took after the destruction of Hirschfeld's research before it was recognized and widely accepted by professionals that pathologizing sexual and gender minorities was counterproductive for community health. Penalizing someone for being gay, bisexual, transgender or gender diverse makes as much sense as punishing someone for something as natural as having red hair. Careful scholarship has found that sexual orientation, gender identity and their expression may manifest at very early ages [3], and that environments that support individuals’ choices regarding how they live their lives result in better health outcomes [4]. In tandem with this awareness, researchers and clinicians have increasingly recognized that sexual and gender minorities have unique healthcare needs, given their diverse behaviours (e.g. different sexual practices), unique clinical exposures (e.g. the use of gender‐affirming hormone therapy) and responses to societal stigma (which may be associated with depression or unhealthy use of recreational drugs for some) [5, 6, 7].

The insights derived from understanding the different clinical needs and outcomes of sexual or gender minority people also led to the recognition that care providers needed specific training to provide optimal care to sexual and gender minority patients. For example, it is clear that many patients will not provide information about their sexual behaviours if providers do not ask, even though most patients (including heterosexuals) are fine with being asked [8]. This can lead to missed opportunities for care and prevention, since many sexually transmitted infections are asymptomatic. Healthcare providers may be the first adults that queer teens can approach to discuss their sexuality or gender identity [9], so it is desirable that they be prepared to give informed and empathic care. In recent decades, the U.S. federal government recognized the need to better understand the health disparities and inequities experienced by sexual and gender minority people by funding peer‐reviewed research proposals that went through the same rigorous application process as other clinical and laboratory research.

But knowledge about appropriate diagnostic tests and medications alone has been shown to be insufficient when addressing sexual and gender minority health. Much like recognition that providers caring for Black patients needed to learn about unique biological issues (e.g. sickle cell disease) as well as culturally specific issues, those caring for sexual and gender minority people needed to understand the role of societal discrimination and stigma in leading to adverse health outcomes. One example of health disparities among sexual and gender minorities has been that they are less likely to undergo cancer screenings, because of anticipated negative experiences when engaging with clinicians [10]. Findings like this provide needed information so that researchers can study the best ways to engage sexual and gender minority people in care and so that health systems can train providers on how to better engage these patients. In the best of all possible worlds, sexual and gender minority health research informs optimal clinical practices for a substantial number of people globally. The most recent Gallup poll found that 7.6% of Americans [11], and 9% of respondents in 30 countries responding to an Ipsos poll [12], identified as a sexual and/or gender minority, so the Trumpian policies may be detrimental to the health of hundreds of millions of people.

It is ironic that it was exactly 100 years ago that Magnus Hirschfeld wrote “Soon the day will come when science will win victory over error, justice a victory over injustice, and human love a victory over human hatred and ignorance.” Unfortunately, that time is not now, reminding us that historical progression is not always an upward curve. Let us hope that the current regression is transient and that the damage can be repaired as soon as possible. But for researchers, clinicians and the communities they serve, until we can effectively counter these ill‐considered actions, the consequences of these malevolent policies may adversely affect millions of lives for decades.

## COMPETING INTERESTS

The author declares no competing interests.

## AUTHORS’ CONTRIBUTIONS

KHM conceptualized and wrote the manuscript.

## Data Availability

The data that support the findings of this study are available on request from the corresponding author. The data are not publicly available due to privacy or ethical restrictions.

## References

[1] Dose R . Magnus Hirschfeld: The origins of the Gay Liberation Movement. New York City: Monthly Review Press; 2014.

[2] accessed April 15, 2025, https://www.hiv.gov/hiv‐basics/overview/data‐and‐trends/statistics.

[3] Pew Research Center . A survey of LGBT American: attitudes, experiences, and values in changing times. Washington, DC: Pew Charitable Trust; 2013.

[4] Valentine SE , Shipherd JC . A systematic review of social stress and mental health among transgender and gender non‐conforming people in the United States. Clin Psychol Rev. 2018;66:24–38. 10.1016/j.cpr.2018.03.003. 29627104 PMC6663089

[5] Makadon HJ , Mayer KH , Potter J , Goldhammer H . The Fenway guide to lesbian, gay, bisexual, and transgender health. Philadelphia, PA: American College of Physicians Press; 2015.

[6] Keuroghlian AS , Keatley J , Shaikh S , Radix AE . The context, science and practice of gender‐affirming care. Nat Med. 2022;28(12):2464–67. 10.1038/s41591-022-02082-w 36522607

[7] Mayer KH , Bradford JB , Makadon HJ , Stall R , Goldhammer H , Landers S . Sexual and gender minority health: what we know and what needs to be done. Am J Public Health. 2008;98(6):989–95. 10.2105/AJPH.2007.127811 18445789 PMC2377288

[8] Cahill S , Singal R , Grasso C , King D , Mayer K , Baker K , et al. Do ask, do tell: high levels of acceptability by patients of routine collection of sexual orientation and gender identity data in four diverse American community health centers. PLoS One. 2014;9(9):e107104. 10.1371/journal.pone.0107104 25198577 PMC4157837

[9] Lung SLM , Wincentak J , Gan C , Kingsnorth S , Provvidenza C , McPherson AC . Are healthcare providers and young people talking about sexuality? A scoping review to characterize conversations and identify barriers. Child Care Health Dev. 2021;47(6):744–57. 10.1111/cch.12892. 34240445

[10] McKay T , Tran NM , Barbee H , Min JK . Association of affirming care with chronic disease and preventive care outcomes among lesbian, gay, bisexual, transgender, and queer older adults. Am J Prev Med. 2023;64(3):305–14. 10.1016/j.amepre.2022.09.025 36460525 PMC10251765

[11] accessed April 15, 2025, https://news.gallup.com/poll/611864/lgbtq‐identification.aspx.

[12] accessed May 6, 2025, https://www.ipsos.com/en/pride‐month‐2023‐9‐of‐adults‐identify‐as‐lgbt.

